# Editorial: Probiotics for nutrition research in health and disease

**DOI:** 10.3389/fmicb.2023.1236442

**Published:** 2023-06-20

**Authors:** José Luiz de Brito Alves, Mariana Monteiro, Helioswilton Sales-Campos, Evandro Leite de Souza

**Affiliations:** ^1^Department of Nutrition, Health Sciences Center, Federal University of Paraíba, João Pessoa, PB, Brazil; ^2^Josué de Castro Nutrition Institute, Federal University of Rio de Janeiro, Rio de Janeiro, RJ, Brazil; ^3^Institute of Tropical Pathology and Public Health, Federal University of Goiás, Goiânia, GO, Brazil

**Keywords:** probiotic, nutrition, gut microbiota, inflammation, oxidative stress

Pathophysiological alteration in the gut microbiota has been linked with developing and maintaining non-communicable diseases. There has been increasing evidence that microorganisms with claimed probiotic properties could benefit human health in different age groups by modulating the host metabolism, physiology, nutrition, and immune functions (Sampaio et al., 2022). Several probiotic strains have been considered safe and commercially available for human use through delivery by foods, beverages, and supplements (de Brito Alves et al., 2020). Additionally, there has been encouraging clinical evidence that some probiotic strains effectively manage and prevent various metabolic disorders and illnesses in humans, characterizing probiotics as promising innovative therapeutic options for their treatment (Cavalcanti Neto et al., 2018; de Brito Alves et al., 2019).

Although several studies have reported that probiotic microorganisms with claimed probiotic properties exert host health benefits, there are current gaps in optimizing the use of probiotics for research nutrition and health that need to be clarified. These have included understanding the mechanisms of how probiotics can affect different biochemical and physiological functions and the optimum dose, frequency, and duration of treatment for different probiotic strains. Additionally, some species-level effects, such as enzymatic activity, bile salt metabolism, neutralization of carcinogen, and vitamin synthesis, or rare strain-specific effects, such as the production of specific bioactive, neurological, immunological, and endocrinological effects have been poorly explored in studies with probiotics. This Research Topic of Frontiers in Microbiology aimed at increasing knowledge on the administration of probiotic microorganisms in the prevention and treatment of chronic diseases, with a particular interest in the underlying cellular and molecular mechanisms. Within this topic, 14 articles have been published and fostered current knowledge regarding the use of probiotics in health and disease.

Several articles on the Research Topic addressed the potential antioxidant and anti-inflammatory effects of potential probiotics strains. The role of *Limosilactobacillus fermentum* HFY06 in preventing colitis was addressed by Liu et al.. The authors isolated the strain from yak yogurt and demonstrated that daily administration of *Limosilactobacillus fermentum* HFY06 at 1.0 × 10^9^ CFU/mL for 2 weeks inhibited inflammation via the NF-kB signaling pathway and prevented the onset and development of colitis in mice. Administering other *Limosilactobacillus fermentum* strain isolated from the traditionally fermented yogurt in China, Liu et al. showed that daily treatment with *Limosilactobacillus fermentum* ZS40 at a low (1.0 × 10^9^ CFU) or high dose (1.0 × 10^11^ CFU) effectively inhibited inflammation via NF-κB signaling pathway and prevented colon cancer in mice. Zhou et al. isolated *Lactobacillus plantarum* GXL94, renamed as *Lactiplantibacillus planturum* GXL94, from fermented chili and showed *in vitro* that this strain has probiotic properties and displayed good tolerance to high concentrations of hydrogen peroxide and strong scavenging capacities to various free radicals, suggesting it to be a potential antioxidant probiotic. Chen et al. showed that daily administration of *Lactiplantibacillus planturum* KSFY, a strain isolated from fermented yak yogurt, at a dose of 1.0 × 10^10^ CFU/kg and 1.0 × 10^9^ CFU/kg for 4 weeks alleviated exercise-induced fatigue and improved exercise capacity in aging mice by ameliorating the metabolite accumulation, glycogen storage, muscle and liver damage, and levels of oxidative stress. Plantaricin BM-1 is a class IIa bacteriocin produced by *Lactiplantibacillus planturum*. After isolating plantaricin BM-1 from *L. plantarum* BM-1, Wang et al. reported a promising anti-cancer effect of this bacteriocin in colon epithelial cells. Mechanistically, the authors showed that plantaricin BM-1 inhibited colorectal cancer by inducing apoptosis via the caspase-3 pathway in colon cancer cells. Lee et al. evaluated the potential anti-inflammatory effects of *Lactobacillus paracasei* ATG-E1, renamed as *Lacticaseibacillus paracasei* ATG-E1, in a mouse model of airway inflammation and respiratory disease. The authors showed that treatment with *Lacticaseibacillus paracasei* ATG-E1 for 12 days improved intestinal barrier function and reduced pro-inflammatory mediators in bronchoalveolar lavage fluid and lung, suggesting that *Lacticaseibacillus paracasei* ATG-E1 may exert preventive and protective effects against respiratory diseases. Considering that probiotic combinations may have superior functions in protecting the gut and regulating the immune system compared to single strains, Li et al. evaluated the effects of a multi-strain probiotic containing *Bifidobacterium lactis* XLTG11, *Lactobacillus casei, Lactiplantibacillus plantarum* CCFM8661, and *Lacticaseibacillus rhamnosus*, named Probio-M9, on ampicillin-induced antibiotic-associated diarrhea in mice. The authors showed that administration of Probio-M9 alleviated ampicillin-induced diarrhea by improving intestinal barrier function, reducing proinflammatory markers, and improving the diversity and composition of the gut microbiota. The production of metabolites or specific bioactive compounds is a strain-specific characteristic of probiotics. Sen et al. performed a comprehensive metabolomic analysis of a co-culture containing *Bifidobacterium breve* MCC1274 and small intestinal-like cells derived from induced pluripotent stem cells (iPS). This co-incubation increased the amount of the immunomodulatory metabolites indole-3-lactic acid and phenyllactic acid in intestinal epithelial cells, which was followed by an upregulation of the expression of genes involved in indole-3-lactic acid synthesis, such as transaminase and tryptophan synthesis-related genes. Postbiotics are bioactive molecules produced by the metabolism of microorganisms that may have health benefits for the host. Abdelazez et al. evaluated the effects of *Levilactobacillus brevis* KLDS, a postbiotic gamma-aminobutyric acid (GABA)-producing strain, against hyperglycemia and hyperlipidemia in streptozotocin-induced diabetes in mice. The authors showed that daily administration of *Levilactobacillus brevis* KLDS at a dose of 250 μL × 10^5^ CFU/ml for 4 weeks reduced hyperglycemia and hyperlipidemia in diabetic mice.

Developing technologies that provide certainty to assess the functional stability of a probiotic product is a challenge for the probiotic industry and companies. The study of Visciglia et al. showed that flow cytometry is a valid, reliable, and innovative tool to assess the numerosity and functional status of a probiotic population compared to the traditional plate count approach.

Han et al. evaluated the effects of the probiotic bacterium *Weissella cibaria* CMU in the treatment of halitosis through a randomized, double-blind, placebo-controlled trial. The authors showed that taking 800 mg probiotic tablets containing 1.0 × 10^8^ CFU for 8 weeks reduced the production of volatile sulfur compounds, hydrogen sulfide, methyl mercaptan, and bad breath in patients with halitosis.

Ramirez-Olea et al. performed a systematic review to demonstrate the potential application of the probiotic *Bacillus licheniformis* as an adjuvant in treating diseases in humans and animals. The authors showed that *Bacillus licheniformis* exerts relevant anti-inflammatory, immunostimulatory, antimicrobial, and antioxidant effects. In addition, *Bacillus licheniformis* had great potential in preventing and treating gastrointestinal, liver, neurological, and cardiovascular diseases, as well as health benefits in growth, dental care, bone health, metabolic, and psychological disorders. Wan and Ma critically revised the efficacy of dietary supplements targeting gut microbiota in preventing and treating gestational diabetes mellitus. The authors showed that the administration of probiotics, prebiotics, and symbiotics is a promising strategy for preventing and treating gestational diabetes mellitus.

Although fermented foods containing live microbes do not fall under the probiotic construct, kefir consumption may have many metabolic health benefits. Bourrie et al. studying cell-free or heat-treated fractions of a pitched kefir showed that the metabolic benefits of consuming this kefir do not require whole kefir.

## Author contributions

All authors listed have made a substantial, direct, and intellectual contribution to the work and approved it for publication.

## References

[B1] Cavalcanti Neto M. P. Aquino J. S. Romão da Silva L. F. de Oliveira Silva R. Guimarães K. S. L. de Oliveira Y.. (2018). Gut microbiota and probiotics intervention: A potential therapeutic target for management of cardiometabolic disorders and chronic kidney disease? Pharmacol. Res. 130, 152–163. 10.1016/j.phrs.2018.01.020 29410236

[B2] de Brito Alves J. L. de Oliveira Y. Carvalho N. N. C. Cavalcante R. G. S. Pereira Lira M. M. Nascimento L. C. P. D.. (2019). Gut microbiota and probiotic intervention as a promising therapeutic for pregnant women with cardiometabolic disorders: Present and future directions. Pharmacol Res 145, 104252. 10.1016/j.phrs.2019.104252 31054952

[B3] de Brito Alves J. L. de Oliveira Y. Sousa V. P. de Souza E. L. (2020). “Probiotics for humans: Current status and future prospects,” in New and Future Developments in Microbial Biotechnology and Bioengineering Trends of Microbial Biotechnology for Sustainable Agriculture and Biomedicine Systems: Perspectives for Human Health (Elservier) 10.1016/B978-0-12-820528-0.00017-X

[B4] Sampaio K. B. Fusco V. de Brito Alves J. L. de Souza E. L. (2022). “Probiotics: Concepts, evolution, and applications,” in Probiotics for Human Nutrition in Health and Disease 1. 10.1016/B978-0-323-89908-6.00019-4

